# What Is Dentistry?

**Published:** 1898-05

**Authors:** 


### Domestic Correspondence.

### WHAT IS DENTISTRY?

Washington, D. C., March 25, 1898.

To the Editor:

Sir,—A trial of some possible importance has been in progress in the Police Court of the city of Washington involving the above question. The patentee of certain attachments for retaining partial and full dentures in the mouth, which it is claimed is an improvement on previous methods, was on trial for "practising dentistry without a license" from the Board of Examiners of the District of Columbia. It developed in the course of the trial that defendant was not a dental graduate. The custom—possibly it is the law—in the District of Columbia is to allow graduates of "reputable" colleges to practise without examination by the board. Persons who are not graduates are required to pass such examination as the board may prescribe. This examination successfully passed, the applicant is furnished with a certificate which gives him license to practise. Without such license he is liable to prosecution for misdemeanor.

Defendant was introduced, or rather introduced himself, to a number of the dental profession of Washington about a year ago, and exhibited his patented appliances in private offices and at public clinics given in the offices of dentists, making such operations in the mouth as were necessary and preliminary to the insertion of the denture. Teeth were implanted, crowns amputated, canals opened and filled, holes drilled in teeth for the insertion of pins, and possibly in some cases fillings inserted for the firm attachment of the fixtures. Quite a number of the profession, taking an interest in the method as shown, had him insert pieces for their patients, he furnishing such materials and fixtures as were needed in his special method. Fees were doubtless taken, but this feature of the case is unimportant. The thing which especially interests the dental profession was the trial which resulted as growing out of the arrest of defendant for practising without a license, as above stated. A large number of dentists of the city were summoned as witnesses, some by the prosecution, some by the defence. Great interest was manifested, for it was felt by not a few that the fate of the law somewhat depended on the outcome. If defendant were acquitted, it was felt that the law was a practical failure, for it was evident that he was not licensed, and also that he had not applied to the Board for such license. Moreover, as stated above, he was not a graduate.

He was, however, a matriculant of a dental college of the District of Columbia, and a good deal of legal fencing was done by counsel to show that what defendant did in a dental way was done as a student of the college, and was thus by the law allowable. The prosecution contended that the work of a student would not be of this character, and drew out of the witnesses for the defence the unwilling admission that the defendant was teaching them (the dentists in whose office he was), not they teaching him; the teaching in question being instruction in the methods used and patented by him.

The principal interest in the case, however, to the profession at large, and that which alone makes this report worth making, is in the "expert" evidence brought out by the defence as to *what constitutes dentistry.*

Possibly no trial, certainly no trial with which the writer is familiar, shows more widely divergent views as to this subject. The border-line between dental surgery and prosthetic dentistry was made the subject of an infinite amount of discussion and hair-splitting wrangling between opposing counsel. Witnesses themselves seemed to have the most opposite and irreconcilable views on the subject. Some seemed positive in the conviction that the two subjects had nothing in common, and that the maker of a "set of teeth," no matter how great his skill, or how difficult the work, was not practising dentistry. Others seemed quite as positive that, in the ordinary acceptation of the word, "dentistry," derived from *dens*, a tooth, meant any work done on a tooth or about it. As an illustration of the one view, one dentist gave as his opinion that the extraction of a tooth, the amputation of the crown, the attachment of a porcelain crown to this root, and the subsequent replacement of the root in the socket from which it had recently been removed was *not* dentistry. It seemed doubtful even to some as to whether the removal of calculus from teeth preparatory to treatment and crowning was dentistry.

The aim of the defence was to prove that what defendant did was not dentistry, the prosecution trying to controvert this. Really the weight of evidence, at least so far as numbers went, seemed rather to show to the jury that what defendant had been doing was not dentistry, although it was proved incidentally that he had filled teeth as well as crowned. A great effort was made to show that crowning, bridge-work, plate-work, etc., was not dentistry.

A leading question, often recurring with the counsel, and about which many questions were asked, was, "What is oral surgery?" In the minds of the greater part of the witnesses there seemed to be a sharp distinction made between oral surgery and dentistry. One witness was understood to say that the only oral surgeon proper in the country was the late Professor Garretson. Nearly all seemed to think that oral surgery was something distinct and but little allied to dentistry. A few thought that any operation in the mouth was oral surgery, whether the extraction of a tooth or the filling of the same, the crowning of a root or the resection of a jaw.

It seems clear, from all the evidence given in this trial, that either the "expert" testimony in this case was not particularly profound, or else that definitions in dentistry need punctuation. What is oral surgery? What is operative dentistry? These certainly are not questions for courts to decide except on expert testimony, and where experts disagree so widely as to what is what, any decision of a court must necessarily be worthless.

One question is of interest. It was clearly shown on the trial that the defendant was skilful. It was shown further that he had invented what was by many believed to be invaluable improvements in dentistry. It was not, I suppose, within the province of the court to decide as to this matter, but the question may come up at any time, and be fought through the courts, What is a man's "vocation"? The constitution protects a man in "the pursuit of his vocation." If dentistry is one's vocation, can any local law interfere with its pursuit? Is not such law unconstitutional? Then may arise that other question to vex the profession and the courts as well, Who is to decide the matter of fitness for a vocation? To illustrate: a college grants a student a diploma. This should be *prima facie* evidence of fitness. The diploma *should*, it would seem, establish the vocation. Has, then, a State board the right or a State legislature the right to pass any law which will interfere with the "vocation" of the holder of the diploma? On the other hand, must the graduate, in addition to what his diploma may say about his fitness for practice, have that fitness further tested by a "board?" These are grave questions, not raised in a spirit of captiousness, but may and probably will arise some day to vex both college faculties and examining boards as well.

One point of interest should be mentioned in connection with the evidence of the "experts." The law is venerable, and precedent counts for almost everything in a court. Because some of the witnesses were men of great age, the judge and jury were urged by counsel to look on such testimony as being peculiarly valuable. As a matter of fact, it may or may not be. It depends. The lines shift so rapidly in dentistry, the work of to-day is so totally different from that of a decade ago, and both progress and variety are so great, as to make it difficult for the keenest-witted and most vigorous-brained young man of the profession to keep the pace. All who know the conservatism of age know that whatever may be the accuracy of judgment of such, the keen, sharp apprehensiveness is dulled by years. This is written in no attempt to derogate from the veneration of age, but as stating a fact that all old men of candor allow.

In conclusion, should this court decide that the operation of attaching a porcelain crown to a natural root,—such a decision is quite possible,—or decide that defendant did not practise dentistry, because he only prepared roots for the attachment of his "patented methods," or that his patents gave him the right to practise, on the principle that the greater must override the lesser, or should a legal distinction be made between oral surgery and operative dentistry (courts have made far more wonderful decisions than such), what effect will all this have on the legal status of dentistry? What effect on the legal status of "Examining Boards"?

Certainly dentistry should have a jurisprudence, but if courts are to decide these things for us, it behooves the profession to look into the matter, that we may not be bound by some decision of a "police court," to be quoted by future courts and lawyers as good law and "expert testimony."

Appended is the decision of the court. The charging of the judge seems clearly to show that in his opinion what defendant did was dentistry, but the jury came to the conclusion that he was not guilty of a violation of the law. How they arrived at such a conclusion is, I dare say, one of the mysteries of the jury system.

James B. Hodgkin.

"The United States branch of the Police Court met this morning at nine o'clock, Judge Scott presiding.

"Judge Scott announced that he had granted prayers offered by Prosecutor Baker, as follows:

"'1. If the jury believe that the defendant has practised or attempted to practise dentistry in the District of Columbia during the period covered by the information or at any time within three years from the filing thereof, and if they further find from the evidence that the defendant has not registered with the health officer, in compliance with the act of June 6, 1892, then they shall declare the defendant guilty, unless they find that the defendant is under *bona fide* pupilage with a registered dentist,—meaning that in good faith the defendant is a pupil or scholar of a registered dentist, or that he is pursuing a regular uninterrupted dental college course and in the course of his studies and practical work in said college.

"'2. The jury are further instructed that it is no defence to the charge contained in the information that the defendant is a student of a dental college in this District, unless they find further that the acts alleged to have been committed by him were committed while he was pursuing a regular uninterrupted dental college course, and in the course of his studies in said college as a part of the course thereof.

"'3. The jury are instructed, as matter of law, that if they believe from the evidence that the placing in the mouth of a denture, or of an anchor-denture, comes within the science of dentistry, then it is no defence to the charge mentioned in the information that the defendant has or claims to have certain patent rights covering a denture.

"'4. The jury are instructed, as matter of law, that if they find from the evidence that the defendant did practise dentistry in the District of Columbia, it is no defence to the charge that he is patentee of certain appliances that are used or placed in the mouth for the purpose of anchoring teeth, and they are further instructed that such patent does not give the defendant a right to practise in the District of Columbia.

"'5. If the jury believe from the evidence that the defendant practised or attempted to practise dentistry in the District of Columbia without having complied with the previous law of dentistry in said District, then the jury are instructed, as matter of law, that it is no defence to the charge against him that he is the owner and patentee of certain letters patent.

"'6. The jury are further instructed that it is no defence to the charge in this information that the defendant is under *bona fide* pupilage with a registered dentist, unless they further find that the acts committed, if they find any acts committed by the defendant, were performed by him in the office wherein he claims to be a pupil, and as a pupil therein.

"'7. If the jury find from the evidence that the defendant merely styles himself a pupil of a registered dentist, and that no *bona fide* pupilage exists, but that he really was the teacher, and if they further find that the defendant has practised or attempted to practise dentistry in the District of Columbia without complying with the act of June 6, 1892, then they shall find the defendant guilty.'

"Three prayers offered by the government were rejected by the court.

"The argument of counsel followed.

"Mr. Jeffords noted an exception to all the prayers offered by Mr. Baker and granted by the court, and gave notice of an intention to apply for a writ of error.

"Judge Scott read to the jury the law in reference to the practice of dentistry in the District of Columbia. He also referred briefly to the evidence, at the conclusion of which the case was given to the jury, who at once retired.

"The jury at 2.22 o'clock this afternoon rendered a verdict of not guilty. The defendant was then discharged."

